# Extraintestinal *Clostridioides difficile* Infections: Epidemiology in a University Hospital in Hungary and Review of the Literature

**DOI:** 10.3390/antibiotics9010016

**Published:** 2020-01-02

**Authors:** Edit Urbán, Gabriella Terhes, Márió Gajdács

**Affiliations:** 1 Department of Public Health, Faculty of Medicine, University of Szeged, Dóm tér 10., 6720 Szeged, Hungary; 2 Institute of Clinical Microbiology, Faculty of Medicine, University of Szeged, Semmelweis utca 6., 6725 Szeged, Hungary; terhes.gabriella@med.u-szeged.hu; 3 Department of Pharmacodynamics and Biopharmacy, Faculty of Pharmacy, University of Szeged, Eötvös utca 6., 6720 Szeged, Hungary; gajdacs.mario@pharm.u-szeged.hu

**Keywords:** *Clostridioides difficile*, extraintestinal, infection, bacteremia, intraoperative infections, abscess, wound infection, epidemiology, anaerobes

## Abstract

Extraintestinal manifestations of *Clostridioides difficile* infections (CDIs) are very uncommon, and according to the literature, poor outcomes and a high mortality have been observed among affected individuals. The objective of this study was to investigate the incidence rate of extraintestinal infections caused by *C. difficile* (ECD) in a tertiary-care university hospital in Hungary. During a 10-year study period, the microbiology laboratory isolated 4129 individual strains of *C. difficile*; among these, the majority were either from diarrheal fecal samples or from colonic material and only *n* = 24 (0.58%) were from extraintestinal sources. The 24 extraintestinal *C. difficile* isolates were recovered from 22 patients (female-to-male ratio: 1, average age: 55.4 years). The isolates in *n* = 8 patients were obtained from abdominal infections, e.g., appendicitis, rectal abscess or Crohn’s disease. These extraintestinal cases occurred without concomitant diarrhea. In all, but two cases *C. difficile* was obtained as a part of a polymicrobial flora. Our isolates were frequently toxigenic and mostly belonged to PCR ribotype 027. Resistance to metronidazole, vancomycin, clindamycin and rifampin were 0%, 0%, 20.5% and 9.7%, respectively. The increasing amount of reports of *C. difficile* extraintestinal infections should be noted, as these infections are characterized by a poor outcome and high mortality rate.

## 1. Introduction

*Clostridioides difficile* (formerly *Clostridium difficile*, *Cd*) is a Gram-positive, spore-forming, anaerobic bacillus, which is commonly found in the intestinal tract of animals and humans and in the environment [1]. The bacteria could produce exotoxins, namely toxin A (tcdA), toxin B (tcdB) and binary toxin (CDT); the toxin-producing strains are the common cause of antibiotic-associated diarrhea (AAD), accounting for 15%–25% of all episodes of AAD [2]. Risk factors associated with transmission are exposure to antibiotics (especially broad-spectrum agents, e.g., amoxicillin-clavulanic acid, clindamycin and fluoroquinolones), gastrointestinal surgery/manipulation, an extended stay in hospital, serious underlying illnesses (e.g., immunosuppression and cancer) and advanced age. In the last several decades, the frequency and severity of intestinal *Cd* infections (CDIs) have been increasing worldwide to become one of the most common and serious hospital-acquired infections (HAIs) [3]. In 2002, an outbreak in Quebec, Canada, demonstrated the emergence of a virulent strain type known as the North American pulsed-field gel electrophoresis type 1 (NAP1 or PCR ribotype 027) [4]. This strain type was associated with an increase in the number of *Cd* outbreaks in hospitals with higher rates of recurrence and mortality and it also stressed the importance of research in the field of microbiology, epidemiology and infection control [5]. *Cd* may be a causative agent in infections outside the intestine, but there is very little information available about extraintestinal infections caused by this pathogen. Nevertheless, with an increase in the incidence of *Cd*-associated colitis, there has also been a similar increase in cases of extraintestinal (or extracolonic) *Cd*.

Extraintestinal CDIs are rare but their clinical role has been reported by Smith and King in 1962 [6], much sooner, than the recognition of the significance of *Cd* in enterocolitis. Most extracolonic infections of *Cd* are preceded by gastrointestinal disease, either *Cd* colitis or surgical and/or anatomical disruption of the colon. In real-life clinical situations, delineating the pathogenic role of *Cd* in an extraintestinal site is often problematic and questionable. Evaluation of the significance of these bacteria is not always straightforward, especially if the strain is found as a part of a mixed infection. The epidemiology of extraintestinal *Cd* infections may vary greatly by region and time period; therefore, the assessment of local/institutional data is essential to evaluate the regional or national situation and to reflect on international data available [7]. With this in mind, the aim of the present study was to perform a systematic analysis of all consecutive extraintestinal CDI cases over a 10-year study period to establish a complete spectrum of infection as well as the clinical significance of *Cd*, and to report all extraintestinal *Cd* isolated in our institution during the last 10 years.

## 2. Results

During the 10-year study period, the Institute of Clinical Microbiology has isolated 4129 individual isolates of *Cd*. Overwhelming majority of these isolates were either from diarrheal fecal samples or from colonic material (*n* = 4104, 99.42%). After our first recorded case of clinically relevant extraintestinal *Cd*, the detailed epidemiological characterization of these infections was performed [8]: only *n* = 24 (0.58%) was isolated from extraintestinal sources (corresponding to 0.003 cases/1000 patients). The number of extraintestinal CDI cases was 2.2 ± 1.3/year (range: 0–4; with highest numbers in 2009 and 2011 [*n* = 4], while no cases were recorded in 2015). Most of the extraintestinal *Cd* containing-samples originated from the Dept. of Surgery (*n* = 12), while *n* = 3 samples had come from the Dept. of Dermatology and Immunology, Dept. of Pediatrics and Dept. of Internal Medicine and *n* = 1 sample from the Dept. of Obstetrics and Gynecology, Dept. of Maxillofacial and Oral Surgery and Dept. of Urology, respectively. The majority of isolates were recovered from superficial and deep wound exudates (*n* = 6 and *n* = 4, respectively) and abscesses (*n* = 6), additionally, *n* = 3 isolated originated from the abdominal cavity or were taken intra-operatively, while an isolate each was also recovered from a blood culture sample, bile sample and a blister fluid (*n* = 3 overall).

The 24 extraintestinal *Cd* isolates were recovered from 22 patients (males and females in equal measure), with an average age of 55.4 years (range: 9 months–84 years); *n* = 10 patients were over 65 years of age. Most of the patients were suffering from co-morbidities and severe underlying illnesses (Table 1) and only *n* = 8 patients were outpatients; four out of eight outpatients were affected by a skin- and soft tissue infection. In the case of eight patients, the focus of infection was abdominal (appendicitis, abscess in the colon and Crohn’s disease), while for seven patients, *Cd* was found in a dermatological abscess or wound. At the time of isolation, none of the patients presented with diarrheal illness, for this reason, clinicians did not request the examination of a stool sample for *Cd*.

In most cases, *Cd* strains were isolated together with other aerobic, facultative anaerobic or obligate anaerobic bacteria (*n* = 20), *Cd* was the only isolated species in *n* = 4 samples only (two individual samples from two different patients, and two individual samples from a third patient), namely a blood culture, an intraoperatively-collected gynecological sample and two samples from a renal abscess. The most frequently detected co-pathogens were *Escherichia coli* (*n* = 6) and *Enterococcus faecalis*/*faecium* (*n* = 6), respectively (Table 1).

Interestingly, in a 38-year-old female patient attending the Dept. of Maxillofacial and Oral Surgery with an infected salivary gland (sample obtained with sterile puncture), *Cd* was found as a member of a complex bacterial flora, while in a 54-year old female patient, *Cd* was the only isolated pathogen in very high CFU/mL, corresponding to a case of pelvic inflammatory disease (PID), where a surgical sample was taken in the context of aberrant uterine and vaginal bleeding. In case of two patients, *Cd* was isolated on two separate occasions: one patient was a 60-year-old male in the ICU where *Cd* was found as a part of a mixed anaerobic flora in two decubitus wound exudates, and a 32-year-old female, where only a ribotype 027 strain was isolated in high colony forming units from two independent samples of a renal abscess (Table 1).

Toxin-production of the respective isolates was determined in 19 cases; 15 out of 19 isolates tested (79%) were toxin A and toxin B-positive. Ribotyping was performed in 14 cases: seven tested isolates (50%) were corresponding to the hypervirulent 027 PCR ribotype, while three strains were of the 198 PCR ribotype, which is very rarely found in other countries, as it is very specific to the region of the study. Antibiotic susceptibility testing was performed in respect to all isolates for metronidazole (MET), vancomycin (VAN), clindamycin (CLI) and rifampicin (RIF). MET and VAN susceptibility was 100% (24/24), resistance rate against and CLI was 20.5% (5/24), while 9.7% for RIF (2/24).

## 3. Discussion and Review of the Literature

The results of our present study highlight already existing data on the infrequent occurrence of the extraintestinal manifestations of CDIs. Extraintestinal *Cd* infections were observed sporadically among patients hospitalized in our university hospital in Szeged between the 1 January 2008 and 31 December 2017. Previously, we reported a rare extraintestinal infection caused by toxigenic *Cd* in a 31-year-old male patient admitted to the hospital, who was a victim of an accident and was subsequently polytraumatized [8]. He injured his lower limbs harvesting potato; thus, his lacerated wounds were grossly contaminated by soil. Microbiological examination of the pus revealed a toxin-producing *Cd* as an etiological agent of this infection: culture from the lateral wound sample showed the presence of a high CFU/mL of a toxin-producing *Cd* strain by anaerobic incubation and few colonies of *Bacillus subtilis* strain by aerobic incubation. After the molecular genetic investigation, the strain proved to be a 014-PCR ribotype and XVIII. toxinotype, A and B toxin-positive, and binary toxin-negative strain by PCR methods and sequencing [8]. Our patient was not immunocompromised; he had no history of diabetes, malignancy and gastrointestinal disorders. Our patient had none of the well-known risk factors of recent hospitalization and broad-spectrum antibiotic use prior to the extraintestinal manifestations; in line with this, there are increased reporting of abscesses caused by this the organism in the absence of diarrhea: this is a concerning finding and may be a sign of the emergence of new toxigenic strains in the asymptomatic carriers.

Extraintestinal infection due to *Cd* is a rare finding and these are usually reported in the form of case reports. Until now, there are only some case reports in the literature: Jacobs et al. described extracolonic *Cd* infections of the small bowel and reactive arthritis [9] and reviewed the literature in 2001. In this publication, they have found that preceding invasive interventions are often associated with the involvement of the small intestine, corresponding to a high mortality rate (4/7 patients), especially if concomitant bacteremia is also present. These infections were usually of polymicrobial nature (associated with the usual microbiome of the colon). Based on existing reports, *Cd*-associated reactive arthritis has the following characteristics: it is unrelated to the presence/absence of the MHC-B27 status of the affected patient, arthritis is generally polyarticular and fever may or may not be present [9,10]. The tibiofemoral joint (in the knees) and the condyloid-type joint in the wrist were the most commonly affected joints [9,10,11,12]. The onset of reactive arthritis was-on average-around eleven days following the occurrence of diarrhea. The disease took around 4–5 weeks to resolve, therefore, it may be considered a prolonged condition [9,10,11]. If diarrheal illness or the isolation of *Cd* does not take place (culture negativity and no clinical suspicion), rare clinical entities in children and adults caused by *Cd* (prosthetic device infections, osteomyelitis, necrotizing fasciitis and cellulitis) may be very difficult to ascertain [9,10,11,12]. Infections associated with skin and bone involvement as usually preceded by some sort of exogenous trauma: it would be reasonable to suspect that the development of extraintestinal *Cds* (ECDs) may be due to the contamination of the infected site by *Cd* spores of environmental or patient-related origin. [9]. It may, however, also cause disease in a variety of other organ systems [10,11,12]. Nevertheless, additional extraintestinal *Cd* (EID) manifestations have also been described: cellulitis [9], prosthetic and periprosthetic joint infections [11,12,13,14], bone infections [15,16], splenic and cranial involvement in the form of abscess formation [17,18,19], pericarditis [20] and infections of the serous membrane of the pleura [21], while case-report-based evidence for fasciitis necrotisans [22,23], visceral abscesses [18], empyema [24] and appendicitis [25] have also been reported in the literature as uncommon presentations of extraintestinal CDIs. Of these extracolonic infections, most were polymicrobial and commonly involved the enteric flora [9,21]. Simpson et al. [24] reported one case of CDI in empyema, where the proposed route of infection was a chest tube, leading into a pre-existing empyema. However, association between stool positivity and clinical presentation could not be established, as no stool samples were sent for culture. [24] The only other mechanism through which the pleural fluid could be contaminated is hematogenous spread [21].

It is important to report that most cases of extraintestinal CDI were polymicrobial in nature, Marina et al. [26] reported on anaerobic pleuropulmonary infections and found that of 116 organisms isolated, only two were *Cd* and only one of these was isolated in a pure culture. Roy et al. [27] described a patient presented with hepatic and pancreatic abscess with a polymicrobial infection. With the findings of sigmoid perforation, they considered the route of infection being translocation of bacteria across the gastrointestinal lumen due to colonic inflammation with direct seeding to the site. Direct spread of *Cd* from the intestine has been reported in previous case reports as well, where intra-abdominal spread of infection was suspected due to transient bacteremia in a case of alcoholic cirrhosis, with *Cd* isolated from ascites fluid, but in this patient, blood cultures remained negative [28]. *Cd* is rarely identified as a cause of pyogenic liver abscess, there are only three reported case of liver abscesses due to *Cd*, Sakurai et al. [29] and Ulger et al. [30] have reported liver abscess due to the organism. In the publication of Sakurai et al., a case of monomicrobial infection in a 75-year-old woman was reported, who had a prior drainage of a hemorrhagic liver cyst and was readmitted 2 years later with fever and no reported diarrhea in the patient [29]. Ulger et al. reported a case of liver abscess in an 80-year-old, non-hospitalized female, who had no reported diarrhea or antibiotic use prior to diagnosis of multiple liver abscesses due to the organism [30]. Morioka et al. [31] described a case of a 74-year-old male with primary biliary cirrhosis and hepatocellular carcinoma, who underwent transarterial chemoembolization (TACE). One month after hospitalization, the patient was re-admitted due to a liver abscess at the same site of the TACE. *Cd* was isolated solely from the liver abscess and both of anaerobic blood cultures were positive after 13-h incubation. At this time GDH and toxin A/B showed positivity in the stool samples; but the patient had no apparent diarrhea since then. Toxin A and B and binary toxin production all of strains isolated from the blood and liver abscess were identified by PCR using nonrepeating sequences of toxins A and B and the repeating sequence of toxin A and *cdt* (binary toxin): all strains tested were positive for toxin A, toxin B and binary toxin [31].

There are only a few retrospective studies involving patients with extraintestinal CDIs at a single institution over the past decade, aiming to better characterize the syndrome and to correctly establish the real incidence. In their first report (chronologically), Smith and King [6] published a total of eight strains of *Cd,* which were isolated from cases of infections in humans from seven different bacteriological laboratories. One of these was isolated from a case of gas gangrene, one from an abscess following a fractured femur, one from a blood culture from an infant, two from pleural fluid, two from peritoneal fluid and one from an abscess in the vaginal vault. According to their findings there was no evidence (in those cases), that *Cd* is pathogenic for man [6]. The role of intermittently occurring *Cd* bacteremia was highlighted as an important factor of transmission from colonic to extracolonic infections such as osteomyelitis, visceral abscess and prosthetic joint infections, however, blood cultures positive for *Cd* have rarely been reported. In 1995, in a report published by Chatila and Manthous [32] including four patients, and three additional cases published by Lowenkron et al. in 1996 [33], sepsis-like condition of the patients were reported, in conjunction with severe pseudomembranous colitis, resulting in the rapid deterioration of the patients; these patients needed mechanical ventilation, administration of broad-spectrum antibiotics and support for hypotension. Although positive blood cultures were not reported, it was suggested that the sepsis-like syndrome in these patients was associated with *Cd,* originating from a cellular inflammatory response to either severe enterocolitis alone or to other factors such as toxin A and B activation of a host monocyte and neutrophilic chemoattractant response [32,33]. Based on these reports, the systemic inflammatory reaction (SIRS) may be due to the diffusion of the bacterial toxins across the damaged gut mucosa (with increased permeability) [32,33]. Fifteen cases of *Cd* bacteremia (published in English by 2009) were summarized by Libby et al. [34]. The first report was in 1962 in a 5-month-old baby presenting with a cough, inflammation of the mucous membranes lining the nasal cavity and loss of appetite [6]. Twelve out of the fifteen cases had presented with Gram-negative and/or anaerobic bacteremia, representing bacteria from the gut flora, while all cases of *Cd* bacteremia in adults had some kind of gastrointestinal illness of varying severity; 12 out of 15 patients reported prior antibiotic exposure. Two patients with polymicrobial *Cd* bacteremia were affected by blood cancer, while one patient had two separate episodes of *Cd* bacteremia, during which the status of the GI-disease of the patient worsened [34]. 7-day mortality was 50% (*n* = 7), two patient outcomes were unknown, while *n* = 5 patients were discharged from the healthcare facility. The results from stool toxin assays were described in only three cases. All but three of the 15 cases of *Cd* bacteremia were polymicrobial, all cases of monomicrobial *Cd* bacteremia were in adult patients with similar symptoms (concomitant fever, abdominal pain and diarrhea) [34]. *Cd* bacteremia was also noted in a female patient (suffering from leukemia), lacking classical gastrointestinal symptoms or radiological findings of colitis or any ongoing gastrointestinal pathology [34]. Dauby et al. recently reported a case of monomicrobial bacteremia due to *Cd* and identified the strain as ribotype 002 [28]. *Cd* bacteremia has been reported in 27 adult patients with gastrointestinal disease since 2011 and most of these cases involved polymicrobial bacteremia [32,33,34,35]. Overall, the mortality rate among patients with *Cd* bacteremia may be estimated to be around 20%–40%, based on published reports [32,33,34,35].

In a review from the United States, identifying seventeen published cases of extraintestinal *Cd* (ECD) infection [36], the bacteria were isolated from the following sample types: pleural fluid, peritoneal fluid, peripheral blood and biopsy of the bones, in addition to abscesses of the spleen, vagina and thigh. In contrast, only in three out of 17 cases were reported, when ECD was associated with the detection of *Cd* toxin in the stool sample. A report from a tertiary-care teaching hospital in Madrid, Spain also identified seventeen cases of extracolonic *Cd* over a 10-year period [37]. *Cd* isolates in twelve patients were originating from samples obtained from close anatomical proximity to the colon. Five cases of peritonitis in five cases (three primary and two secondary), four cases of intra-abdominal abscesses and four cases of abdominal wound infections were the most significant finding, while five other patients had infections not in anatomic vicinity of the large intestine (a brain abscess, two bacteremic episodes and two cases of infected lower limbs) [37]. Most of the reported isolates were non-toxigenic (verified by the fact that diarrhea was not reported in these patients during these pathologies) and patients had no prior antibiotic therapy in their anamnestic data; 9/17 (53%) of patients survived [37]. Nevertheless, death could not be directly attributed to *Cd* in any of the cases. In another paper from the Alabama, USA [38], fifty-nine cases of extraintestinal *Cd* infections were reported that might be grouped into three main clinical presentations: bacteremia (in the presence or absence of evidence regarding focal infections), intra-abdominal infections and abscess formation outside of the abdominal cavity. *Cd* strains were isolated as a part of a polymicrobial flora in the majority (68%) of cases (mainly associated with bacteremia or abscesses, in 40/59 cases) [38]. There is evidence that *Cd* has the ability to potentate infections in polymicrobial infectious processes [38]. While more than two-thirds of affected patients presented with serious underlying conditions (e.g., cancer and renal failure) or interventions, ~30% did not have any of the specific predisposing factors. It has been hypothesized that after the damage and breakdown of the physiological mucosal barrier in the intestines, Cd may gain access to the systemic circulation or the abdominal cavity. However, the interpretation of this phenomena remains difficult, as presented in the publication, GI symptoms were not present (16 cases) or not specifically reported (17 cases) in a high number of patients. Likewise, diarrheal illness was not reported in 18/59 cases with extraintestinal infection; overall mortality in this paper was reported to be 37% [38]. A significant drawback of most of the reports detailing ECD cases is that toxin-production assays (which may be considered the principal virulence factor for *Cd*) are infrequently performed; however, in the relatively low amount of papers where this data is available, these infections are usually caused by non-toxigenic strains. A landmark study from Minneapolis, USA [39] reported that the levels of immunoglobulin G antibodies against toxin A was 20-fold higher, in contrast, levels of immunoglobulin A antibodies were three-fold higher in a patient who developed a splenic abscess due to a *Cd* infection, compared to the mean levels of the “control” group of patients (*n* = 14), who presented with *Cd* diarrhea only. However, the role and clinical significance of these naturally occurring antibodies during the progression of the ECD infections is not yet known [39]. Visceral abscess formation is usually due to hematogenous spread of microorganism, mainly involving the spleen, with one reported case of pancreatic abscess formation [17,39]. Frequently, these abscesses are only recognized weeks to months after the onset of diarrhea or other colonic symptoms [17,18,39].

In a 10-year retrospective, single-center survey performed by Mattila et al. [40], only 0.17% of the CDI cases were reported to be extraintestinal. As reported in their study, 81% of the cases were inpatient and had received antibiotics, which increased the risk of infection. ECD was found in 31 patients: two patients had bacteremia, four had abdominal infections without any prior surgery, seven had abdominal infections after surgery, four had a perianal abscess, 13 had wound infections and one had *Cd* in a urinary catheter [40]. From the Mayo Clinic Rochester, Gupta et al. [41] reported their single-center experience in evaluating the clinical burden of ECD, and they have also characterized the clinical management and patient outcomes of these infections. A retrospective review of available medical records was conducted, identifying patients with *Cd* from extraintestinal sites between 2004 and 2013; overall, 40 patients with extraintestinal CDI were identified, out of which twenty-five had abdomino-pelvic infections, eleven had bacteremia, three had wound infections and one had a pulmonary infection [41]. *Cd* was isolated alone in 37% of cases, with 85% of infections being reported of nosocomial origin. Indicators associated with the high-risk for extraintestinal CDI infections identified in this paper were the following: GI surgery (88%), recent use of broad-spectrum antimicrobials (88%), cancer (solid tumors; 50%) and the use of drugs affecting the GI tract (i.e., H^+^/K^+^ ATPase-inhibitors, 50%). Loose stools were recorded in 18/40 patients, while in 12/40 presented with fecal samples PCR-positive for *Cd*. Resistance to metronidazole and piperacillin-tazobactam in these isolates were 0% [41]. In this report, 39/40 patients received antibiotics, in addition to guided drainage of the infection site; during the study Gupta, et al. reported a mortality rate of 10/40 patients (25%), the death of the patients occurred at a median of around two weeks (range: 1–61 days), after the subsequent isolation of *Cd* [41].

All of the abovementioned studies are in agreement regarding the identified risk factors for extraintestinal CDIs, namely the immunocompromised status of the patient, surgical manipulation of the gastrointestinal tract, recent multiple- or broad-spectrum antibiotic exposure and recent hospitalization. However, it is also noteworthy that except for cases involving the small intestine and reactive arthritis, most of the cases of extracolonic *Cd* disease did not appear to be strongly related to previous antibiotic exposure. In addition to these well-known risk factors, Mutlu et al. [42] also reported the alteration of the colonic microbiome in alcoholics, which was noted to be similar to the enteritis due to *Cd* colitis, suggesting alcohol abuse as a possible risk factor for ECD [42]. As seen in the previous studies, there are some reports available on extraintestinal *Cd* infections, however, the extension of the infection beyond the intestinal tract has not been well understood; therefore, the organism at an extraintestinal site is often a surprise. In addition, extraintestinal *Cd* infections are usually found in conjunction with other microorganisms [9,10,11,12,13,14,15,16,17,18,19,20,21,22,23,24,25,26,27,28,29,30,31,32,33,34,35,36,37,38,39,40,41,42]. The increasing evidence and amount of reports of extraintestinal infection due to *Cd* should be seen as a warning sign, as these uncommon infections are characterized by poor outcome with a high mortality rate. The therapy of extraintestinal CDIs is similar to those of enteric infections (antimicrobial therapy) in combination with surgical debridement, if necessary [9,10,11,12,13,14,15,16,17,18,19,20,21,22,23,24,25,26,27,28,29,30,31,32,33,34,35,36,37,38,39,40,41,42]. There were several reports on extraintestinal CDI cases, where non-toxin producing strains were presenting as significant pathogens, therefore, suggesting that unspecified virulence factors may be important in promoting extraintestinal infection by this microorganism. Therefore, further studies are indicated to better identify these potential virulence factors.

The comparative analysis of the antimicrobial susceptibility and toxin-production of *Cd* isolates of extraintestinal and intestinal origin was not a part of this present study; in addition, our institution does not perform routine susceptibility-testing from diarrheal *Cd* isolates. Toxin-positivity of stool samples received by the institute during the study period was between 20% and 25% (unpublished data). However, over the 10-year period, susceptibilities of diarrheal *Cd* isolates were studied in epidemiological studies in Szeged and other cities in Hungary, spanning a shorter time periods. Between 2006 and 2007, laboratories from West, Central and Southeast Hungary collected *n* = 150 *Cd* isolates from diarrheal feces [43]: 80% of isolates were positive for toxin A and toxin B (this number was 70.5% between 2002 and 2003 [44]), while resistance rates were 0%, 6.3%, 25.0% and 37.5% for MET, RIF, moxifloxacin (MOX) and erythromycin (ERI), respectively [43]. In a study spanning between 2008 and 2010, the resistance levels of *n* = 188 toxin-producing Cd isolates (originating from three centers in Hungary, including Szeged) were 0%, 0%, 14.9% and 19.7% for MET, VAN, RIF and MOX, respectively [45]. In addition, fidaxomicin susceptibilities were also tested, and the majority of isolates (97.9%) exhibited low MIC values. In the same time period, the results of a different study, reporting on the susceptibility of non-027 PCR ribotype strains (to avoid over-representation of this outbreak strain) showed resistance levels to be 0%, 21.5%, 29.5% and 31.0% for MET, MOX, CLI and ERI, respectively [46]. While between the first two study periods (2002–2003 vs. 2006–2007), an increasing tendency in the MICs was recorded, such increase was not observed between the second and third period (2006–2007 vs. 2008–2010) [43,44,45,46].

## 4. Materials and Methods

### 4.1. Study Design and Data Collection

This retrospective observational study was performed on the basis of microbiological data collected regarding a 10-year time frame (from 1 January 2008 to 31 December 2017). The Institute of Clinical Microbiology is the dedicated microbiological diagnostic laboratory of an 1820-bed tertiary-care university-teaching hospital in Szeged, Hungary. This clinical center is responsible for the medical care of over 600,000 patients in the southeast region of Hungary (population: around 1.3 million people based on the most recent census data) [47,48]. Additionally, during the study period, the Institute was also working as the National Reference Laboratory of Human Pathogenic Anaerobic Bacteria in Hungary. Data were collected by an electronic search of the Institutional laboratory information system (LIS) records for the designated time period. The inclusion criterion for analysis was the detection of *Cd* from an extraintestinal sample in the abovementioned study period (2008–2017) [37]. Polymicrobial infection was defined as the isolation of more than one organism in a single sample. As a part of this study, data on the affected patients were also collected, which was limited to their demographic characteristics only (age, sex and inpatient/outpatient status), the basic the indication for sample submission, underlying conditions and the sample origin [48]. The study was deemed exempt from ethics review by the Institutional review board and informed consent was not required as data anonymity was maintained.

### 4.2. Identification of Isolates

*Cd* strains were isolated from extraintestinal sites using conventional, routine methods in anaerobic bacteriology, according to standard descriptions [49,50]. These methods were not specifically designed to detect *Cd* strains only, but rather to detect all of the culturable anaerobic bacteria in these samples [51]. Sample preparation and microbiological culture were carried out according to the routine microbiological practice [49,50]. Briefly, samples were suspended in 1 mL of pre-reduced brain–heart infusion broth (BHI, with a pH set at 7.2; Oxoid, Basingstoke, UK), which was plated immediately on the appropriate selective and non-selective culture media. The following media was used for the quantification of total cultivable facultative and anaerobic bacterial flora: Columbia agar base (Oxoid, Basingstoke, UK) supplemented with 5% (*v*/*v*) defibrinated bovine blood, hemin and vitamin K_1_ [8,49]. The incubation for the isolation of aerobic bacteria was carried out at 37 °C in a 5% CO_2_-containing environment for 48 h. For the isolation of anaerobes, bacterial cultures were set up and incubated in an in an anaerobic environment (containing an atmosphere of 90% N_2_, 5% H_2_ and 5% CO_2_; (Baker Ruskinn, York, UK) for 2–5 days at 37 °C. Isolation of *Cd* from peripheral blood samples was performed using the BacT/ALERT Culture Media system (bioMérieux, Marcy-l’Étoile, France). Isolation of *Cd* in stool samples was carried out using *Cd*–selective Brazier CCEY agar (cefoxitin-cycloserine-egg yolk-agar, Mast Diagnostics, Bootle, UK) at 37 °C for 42 h in an anaerobic atmosphere. Colonies with typical morphology, fluorescence and odor were presumptively identified as *Cd*, which was verified by biochemical and MALDI-TOF MS [8,49].

During the identification of the isolates, Gram-staining and growth requirements for each individual colony type (identified phenotypically using a colony microscopy) provided the basis for choosing the relevant biochemical identification tests to be used. The identification of bacterial and fungal isolates was carried out using classical biochemical tests and ATB/VITEK (bioMérieux, Marcy-l’Étoile, France) and matrix-assisted laser desorption/ionization time-of-flight mass spectrometry (instrument: Microflex MALDI-TOF MS Biotyper (Bruker Daltonics Gmbh., Bremen, Germany) software: MALDI Biotyper Library 3.1); the sample-preparation methods and the technical details of the measurements were performed as previously described [52].

### 4.3. Determination of Toxin Production and Ribotyping, Antibiotic Susceptibility Testing

Toxin production of the isolates was tested using an immunochromatographic method, detecting toxin A and toxin B (*C. difficile* QuikChek Complete, Techlab, Blacksburg, VA, USA). Samples with a GDH-positive, but toxin A/B negative results were further tested with the TC (toxigenic culture) method [44]. Strain typing was performed by PCR ribotyping method as described by Stubbs et al. [53].

Metronidazole (MET), vancomycin (VAN), clindamycin (CLI) and rifampicin (RIF) susceptibility of the isolates were tested using the E-test methodology (AB BIODISK, Solna, Sweden). A *Cd* suspension (of 0.5 McFarland turbidity) of each strain was swabbed on Brucella agar (Oxoid, UK) supplemented with 5% defibrinated sheep blood, 1 mg/L hemin and 5 mg/L Vitamin K_1_ [50]. E-test strips of MET, VAN, CLI and RIF were applied onto the agar surface and the plates were incubated in an anaerobic atmosphere for 24–48 h. MICs were read at the point at which the zone of complete inhibition intersected the MIC scale. For the evaluation of the susceptibility of the tested isolates, EUCAST breakpoints and epidemiological cut-off values (in case of RIF) were used (http://www.eucast.org). *C. difficile* ATCC 9689, *C. perfringens* ATCC 13,124, *B. fragilis* ATCC 25,285 and *C. acnes* ATCC 11,827 were used as control strains.

## 5. Conclusions

Results from our 10-year retrospective study further highlight the low incidence of extraintestinal CDI (0–4 cases/year in our settings), corresponding to 0.58% of total isolations of *C. difficile* between 2008 and 2017. Their isolation in from extraintestinal samples was frequently associated with gastrointestinal tract surgery and other well-known risk factors for intestinal CDI. Interestingly, 36.7% of the patients were outpatients and four of them had some form of skin and soft tissue infection. In most cases, *C. difficile* strains were members of the mixed aerobic/anaerobic flora, with only *n* = 4 specimens where the *C. difficile* strain was the sole pathogen. The most common co-pathogens were *E. coli* and *E. faecalis*/*faecium*, while only four anaerobic pathogens were present as co-pathogens with *C. difficile*. The toxin production of the strains was determined in 19 isolates and 79% proved to be a toxin A and B-producing strain, while for 50% of the strains, ribotyping was also performed and three strains were identified as the rare, 198 (Hungarian: Szeged) PCR ribotype. Antibiotic susceptibility of the strains was high to the tested antibiotics. Although the number of isolates were low during the study period (similarly to other publications in the literature), the present paper reports on a decade’s worth of data from a tertiary-care teaching hospital, in addition to presenting a literature summary of the available references on the topic.

## Figures and Tables

**Table 1:** Data of patients with extraintestinal *Cd* infections, 2008–2017.

Year of Isolation	Dept. or Ward	O/I	Sex	Age	Disease/Indication for Sample Submission	Sample of Interest	A/B Toxin	RT	Co-Pathogens Isolated
**2008**	Surgery	I	M	82	Acute appendicitis	Surgical sample	+	078	*E. faecium*
									*Veillonella* spp.
									*Candida tropicalis*
	Pediatric ICU	I	F	9 months	Invagination of the bowel	Puncture for abdominal cavity	+	011	*Bacteroides vulgatus*
									*B. uniformis*
									*C. ramosum*
**2009**	Maxillofacial and Oral Surgery	O	F	38	Inflammation of the salivary gland	Abscess	−	n.d.	*Streptococcus viridans*
									*Campylobacter ureolyticus*
									*Prevotella buccae*
									*Peptostreptococcus anaerobius*
									*Veillonella* spp.
	Urology	O	F	37	Inflammation of the skin and subcutaneous tissue	Superficial wound exudate	+	198	*Actinomyces meyeri*
									*C. ureolyticus*
									*P. anaerobius*
									*P. melaninogenica*
	Surgery	I	M	69	Gangrene	Surgical sample	−	n.d.	*A. prevotii*
									*P. buccae*
									*P. intermedia*
	Dermatology	O	M	60	Varicose veins of the lower limbs with ulceration	Abscess	−	n.d.	*S. pyogenes*
									*Staphylococcus aureus*
									*C. ureolyticus*
									*Cutibacterium acnes*
									*Eubacterium limosum*
									*Craterellusfallax*
**2010**	Surgery	I	M	58	Gallstone disease	Bile	+	027	*C. bifermentas*
	OB/GYN	I	F	54	Abnormal uterine and vaginal bleeding	Deep wound exudate	+	198	-
	Dermatology	O	M	22	Erythema intertrigo	Superficial wound exudate	−	488	*S. aureus*
									*P. anaerobius*
									*C. ureolyticus*
									*E. lentum*
									*Mobiluncus* spp.
**2011**	Cardiology	I	F	80	Disorders of the electrolyte balance	Blood culture	+	027	-
	Surgery	O	F	22	Rectal abscess	Abscess	+	027	*C. albicans*
									*E. coli*
									*S. pyogenes*
									*P. bivia*
									*P. oralis*
									*P. anaerobius*
									*E. limosum*
	Pediatrics	O	M	17	Therapy-resistant Crohn’s disease	Abscess	+	018	*S. acidominimus*
									*E. coli*
									*P. anaerobius*
									*Finegoldia magna*
									*C. bifermentans*
									*P. melaninogenica*
	Surgery	O	F	80	Abdominal pain	Surgical sample	+	027	*S. pyogenes*
									*Morganella morganii*
									*P. anaerobius*
									*B. fragilis*
**2012**	ICU	I	M	84	Septicemia	Blister fluid	n.d.	n.d.	*Acinetobacter* spp.
									*C. acnes*
	Surgery	I	M	70	Malignant tumor of the flexura lienalis	Surgical sample	+	027	*E. coli*
									*E. faecium*
									*Pseudomonas aeruginosa*
									*E. lenta*
									*B. fragilis*
**2013**	ICU	I	M	60	Myasthenia gravis, chronic kidney failure	Deep wound exudate (*n* = 2)	n.d.	n.d.	*P. mirabilis*
									*P. melaninogenica*
									*B. fragilis*
									*B. ovatus*
	Surgery	I	F	32	Abscess in and around the kidneys	Abdominal puncture sample (*n* = 2)	+	027	-
**2014**	Internal Medicine	I	F	81	Ventricular fibrillation and flutter	Abscess	n.d.	n.d.	*E. coli*
									*E. faecalis*
									*A. vaginalis*
									*B. uniformis*
	Surgery	I	M	68	Localized infections of the skin and subcutaneous tissue	Deep wound exudate (*n* = 2)	+	198	*P. mirabilis*
									*E. coli*
									*E. faecalis*
									*F. magna*
									*P. anaerobius*
									*B. fragilis*
**2016**	Dermatology	O	F	80	Ulceration of the lower limbs	Superficial wound exudate	+	n.d.	*P. aeruginosa*
									*S. dysgalactiae*
									*P. anaerobius*
									*Lactobacillus* spp.
**2017**	Surgery	I	M	40	Unknown disease of the pancreas	Abdominal puncture sample	+	027	*E. coli*
									*E. faecium*
									*S. aureus*
									*B. vulgatus*
									*P. merdae*
	Surgery	I	F	83	Localized infections of the skin and subcutaneous tissue	Superficial wound exudate	+	n.d.	*E. faecalis*
									*F. nucleatum*
									*C. histolyticum*
									*A. vaginalis*

Legend: O: outpatient; I: inpatient; ICU: intensive care unit; OB/GYN: obstetrics and gynecology; RT: ribotype; n.d.: not determined; +/−: presence/absence of toxins.
